# Supplementary material for: A UMLS-based spell checker for natural language processing in vaccine safety
Source: BMC Med Inform Decis Mak. 2007 Feb 12;7:3. doi: 10.1186/1472-6947-7-3 (PMC1805499; doi:10.1186/1472-6947-7-3)
Supplement: Additional file 13 — RAP application directory. Sets up directory for RAP (RDF application for PHP) source code [file 1472-6947-7-3-S13.gz › rap/doc/phpdoc/__filesource/fsource_dataset__datasetDataset.php.html]

xml version="1.0" encoding="iso-8859-1"?


File Source for Dataset.php


# Source for file Dataset.php

Documentation is available at Dataset.php

```
1. <?php
2. // ----------------------------------------------------------------------------------
3. // Class: Dataset
4. // ----------------------------------------------------------------------------------


8. /**
9. * Dataset implementation.
10. * Superclass of datasetMem and datasetDb which contains shared functionality.
11. *
12. * <BR><BR>History:<UL>
13. * <LI>05-02-2005                : First version of this class.</LI>
14. *
15. * @version  V0.9
16. * @author Daniel Westphal (http://www.d-westphal.de)
17. * @author Chris Bizer <chris@bizer.de>
18. *
19. * @package     dataset
20. * @access    public
21. ***/

23. class Dataset
24. {
25. /**
26. * Load a Dataset from File
27. *
28. * @param string
29. * @access    public
30. */
31. function loadFromFile($file)
32. {
33. $parser= new TriXParser(&$this);
34. $parser->parseFile($file);
35. }

37. /**
38. * Load a Datset from a string
39. *
40. * @param string
41. * @access    public
42. */
43. function loadFromString($string)
44. {
45. $parser= new TriXParser(&$this);
46. $parser->parseString($string);
47. }

49. /**
50. * Serialize the Dataset to File
51. *
52. * @param  string
53. * @access    public
54. */
55. function serializeToFile($fileName)
56. {
57. $serializer= new TriXSerializer(&$this);
58. $serializer->serializeToFile($fileName);
59. }

61. /**
62. * Serialize the Dataset to string
63. *
64. * @return string
65. * @access    public
66. */
67. function serializeToString()
68. {
69. $serializer= new TriXSerializer(&$this);
70. return $serializer->serializeToString();
71. }
72. }
73. ?>
```

Documentation generated on Thu, 7 Jul 2005 13:41:09 +0200 by phpDocumentor 1.3.0RC3
